# Clinical Trial Awareness, Perceptions, and Participation Among Cancer Patients in Saudi Arabia

**DOI:** 10.3390/healthcare13091044

**Published:** 2025-05-01

**Authors:** Maryam Alotaibi, Laila Alsolami, Sarah AlHarbi, Amal Alagha, Lina Alqurashi, Rahaf Badr, Nouf Almatrafi, Ghada Aladwani, Amal Alotaibi, Alaa Shahbar, Mohammed Alnuhait

**Affiliations:** Pharmaceutical Practices Department, College of Pharmacy, Umm Al-Qura University, Makkah 21955, Saudi Arabia

**Keywords:** clinical trial, cancer, patients, awareness, Saudi Arabia, barriers

## Abstract

**Background:** Clinical trials are essential tools for advancing cancer treatment, improving survival rates, and introducing innovative therapies. However, patient participation in clinical trials remains low in the Middle East, including Saudi Arabia. This study explores Saudi cancer patients’ awareness, perceptions, and willingness to participate in clinical trials while identifying key barriers to enrollment. **Methods:** A cross-sectional survey was conducted using a self-administered questionnaire distributed via email and social media. Saudi cancer patients aged 14 and older, fluent in Arabic or English, and with a confirmed cancer diagnosis were eligible. Ethical approval was obtained, and participation was voluntary with ensured data confidentiality. The survey assessed awareness of clinical trials, perceived benefits, willingness to enroll, and barriers to participation. Data were analyzed using SPSS version 23, with chi-square tests used to examine associations. **Results:** A total of 201 participants completed the survey. Most were female (69.2%), with a mean age of 39.5 years. Awareness of clinical trials was moderate (51.2%), with social media and healthcare providers being primary information sources. While 57.2% recognized the benefits of clinical trials, 44.8% expressed concerns about risks. Trust in healthcare providers influenced willingness to participate, with 49.8% confident in their physician’s recommendations. The most common barriers were fear of side effects (82.6%) and lack of information (42.8%). Only 5.5% had prior experience with clinical trials, but 35.3% expressed willingness to enroll if recommended by a healthcare provider. Gender and income were significantly associated with perceptions and willingness to participate. **Conclusions:** Findings highlight the need for targeted educational initiatives to improve awareness and trust in clinical trials among Saudi cancer patients. Addressing key barriers, particularly concerns about side effects and limited information, could enhance participation, ultimately advancing cancer research and treatment in the region.

## 1. Introduction

Clinical trials are essential in bridging advancements in cancer medical research with improved patient care [1]. They play a pivotal role in identifying and developing effective cancer treatments, having contributed to increased survival rates over the past four decades through earlier detection and novel therapies [2]. With significant progress in understanding the molecular biology of cancer, numerous targeted therapies have been introduced and are continuously evaluated in clinical trials [3]. Participation in clinical trials provides patients with access to the latest and most advanced treatment options, making them particularly important in oncology [3]. While clinical trials have revolutionized cancer treatment worldwide, participation remains limited in the Middle East, including Saudi Arabia [4]. (According to the U.S. National Institutes of Health’s clinical trials registry, only 4% of registered trials are conducted in the Middle East [5].

Among patients with advanced cancer, physicians may recommend clinical trials as a treatment option, yet participation rates remain low, ranging from 2% to 7% globally, despite their potential benefits [6,7]. Several studies have explored the reasons behind low enrollment, identifying patient-related, physician-related, and protocol-related barriers. These include lack of awareness, negative perceptions, financial burdens, logistical challenges, limited trial accessibility, and physician hesitancy in recommending trials [6,7]. In Saudi Arabia, studies have highlighted a lack of culturally relevant information on clinical trials, leading to misunderstandings and reduced willingness to participate [4,5,8]. Patient-related barriers are particularly significant, with factors such as limited knowledge, misconceptions, lack of family support, and concerns about side effects discouraging participation [5]. While many patients express willingness to enroll when informed, significant barriers persist, including fear of side effects, logistical challenges, and lack of physician recommendations [9]. Several studies have explored factors influencing clinical trial participation. Key determinants include trust in healthcare providers, family support, and the economic burden of cancer care [10]. Patients with prior clinical trial experience exhibit greater willingness to participate, as demonstrated in previous studies [11]. Physician recommendations also play a crucial role in influencing patient decision-making [12]. Additionally, participation rates increase during disease progression relative to those at initial diagnosis [13]. (Given these challenges, it is crucial to fully understand the barriers hindering trial enrollment to enhance participation rates. This study assesses awareness, perceptions, and willingness to participate in clinical trials among Saudi cancer patients. By identifying key barriers, this research aims to inform targeted interventions that enhance patient awareness, improve accessibility, and foster a more informed approach to clinical trial participation in Saudi Arabia. Despite global efforts to promote clinical trial participation, data from Middle Eastern countries, particularly Saudi Arabia, remain scarce. Most existing studies are limited in scope or focused on general public awareness rather than the cancer patient population. This gap limits the ability of healthcare providers and policymakers to develop targeted interventions. Therefore, this study aims to assess awareness, perceptions, and willingness to participate in clinical trials among cancer patients in Saudi Arabia, and to identify key barriers and potential facilitators to improve trial engagement.

## 2. Methods

### 2.1. Study Design

This cross-sectional survey was conducted in Saudi Arabia between October 2023 and May 2024 to evaluate cancer patients’ awareness, perceptions, and willingness to participate in clinical trials. A self-administered electronic questionnaire was developed using Google Forms and distributed online through social media platforms, including WhatsApp, Twitter, and Telegram, as well as through various Saudi cancer organizations. Ethical approval was obtained from the Institutional Review Board (IRB) at Umm Al-Qura University, Makkah (Approval No. HAPO-02-K-012-2023-10-1778).

### 2.2. Participants

Eligible participants were adult cancer patients (aged 14 years or older) with a confirmed cancer diagnosis and proficiency in either Arabic or English. Patients who declined participation were excluded from the study. Informed consent was obtained from all participants before they completed the survey.

### 2.3. Questionnaire

The survey was designed to assess patients’ awareness, perceptions, and willingness to participate in clinical trials. It consisted of three main sections. The first section collected sociodemographic data, including gender, age, marital status, education level, employment status, monthly household income, province, and cancer type. The second section focused on awareness and perception of clinical trials, covering aspects such as perceived risks and benefits, trust in healthcare providers, expectations from clinical trials, overall understanding and knowledge, contributions to medical science, ethical considerations, sources of information, willingness to participate, preferred communication channels, support systems, and barriers to participation. The final section explored factors influencing patients’ perceptions, willingness to participate, and awareness of clinical trials. To ensure the validity and reliability of the questionnaire, we conducted a content validity assessment with input from oncology pharmacists, clinical researchers, and patient advocates. A pilot study was then conducted with 15 cancer patients to evaluate the clarity, relevance, and comprehensibility of the questions. The final version of the questionnaire demonstrated strong internal consistency, with a Cronbach’s alpha of 0.85. Feedback from the pilot study was used to refine ambiguous items, ensuring both clarity and relevance.

### 2.4. Statistical Analysis

#### 2.4.1. Population and Sample Size

Between October 2023 and May 2024, we conducted a cross-sectional survey targeting cancer patients across Saudi Arabia to assess their awareness and willingness to participate in clinical trials. Based on a five-year cancer prevalence of 94,951 cases we estimated that a sample size of 385 participants would provide a 95% confidence level with a 5% margin of error. Despite distributing electronic questionnaires via social media and collaborating with Saudi cancer organizations, we received 201 responses [14]. This shortfall may be due to factors such as limited digital literacy, the physical and emotional burden of treatment, privacy concerns, and challenges in effectively reaching the target population. Although our sample size was below the target, it still offers valuable insights into the perspectives of cancer patients in Saudi Arabia regarding clinical trials. To mitigate potential biases associated with the smaller sample, we employed robust statistical methods and accounted for these limitations in our analysis. Future studies might benefit from combining online surveys with in-person interviews to enhance participation rates and gather more comprehensive data. Although our sample size did not reach the target sample of 385 participants, it offers exploratory insights that contribute meaningfully to the understanding of clinical trial perceptions among Saudi cancer patients. This smaller sample may limit generalizability, but we mitigated this by applying robust statistical methods. For future research, we recommend using mixed-method approaches (e.g., combining digital outreach with hospital-based recruitment) and offering logistical support to increase participation.

#### 2.4.2. Data Collection Method

Survey responses were collected and organized in an Excel spreadsheet before being imported into IBM SPSS Statistics version 25.0 (IBM Corp., 2017, Armonk, NY, USA) for analysis.

#### 2.4.3. Variables and Their Measurement

Statistical analyses were conducted using both descriptive and inferential methods. Qualitative variables were summarized as frequencies and percentages. A significance level of *p* < 0.05 was set for all statistical tests.

## 3. Results

This study involved 201 cancer patients in Saudi Arabia to evaluate their awareness, perceptions, and willingness to participate in clinical trials. The participants had a mean age of 39.5 ± 14.9 years, ranging from 14 to 83 years. The majority were female (69.2%), and more than half (53.7%) were married. In terms of education, 46.8% held a bachelor’s degree, while 28.4% had completed secondary education. Employment status varied, with 31.8% employed full-time and 29.9% unemployed. Monthly household income also differed, with 33.8% earning between 6000 and 15,000 SAR, while 31.3% earned below 6000 SAR. Geographically, the highest proportion of participants were from Makkah (41.3%), followed by Riyadh (27.9%). Breast cancer was the most commonly reported type (32.3%), followed by lymphoma (17.9%). A detailed summary of demographic characteristics is presented in Table 1.

### 3.1. Awareness and Perceptions of Clinical Trials

More than half of the participants (51.2%) were aware of clinical trials. Among those aware, social media (24.4%) and healthcare providers (20.9%) were the primary sources of information.

**Figure 1:**
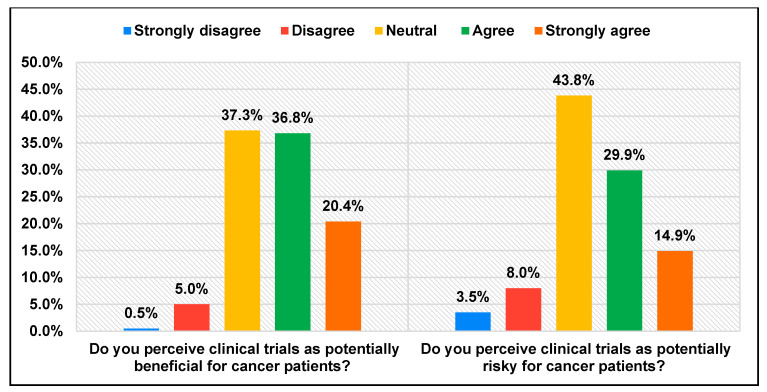
Distribution of the studied participants regarding perceptions of clinical trials.

**Figure 2:**
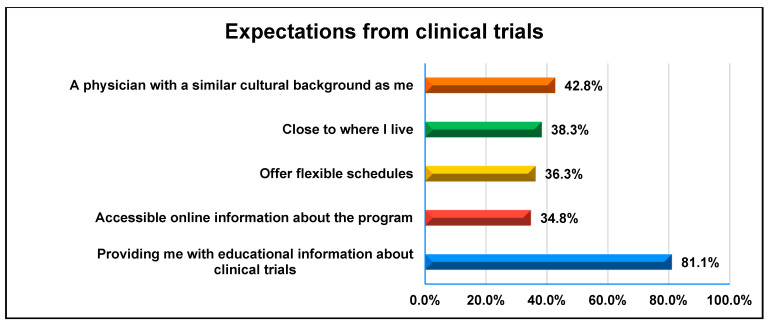
Distribution of the participants regarding expectations from clinical trials.

While 57.2% of participants perceived clinical trials as beneficial, 44.8% viewed them as potentially risky, as shown in Figure 1. Physician trust plays a critical role in clinical trial enrollment. Nearly 49.8% of participants expressed confidence in their physician’s recommendations for clinical trials, while 75.6% felt comfortable discussing questions about clinical trials with their healthcare provider. Participants prioritized several factors when considering clinical trials. Figure 2 demonstrates that the majority (81.1%) expected a comprehensive educational program to enhance their understanding. Additional expectations included access to online trial information (34.8%), flexible scheduling (36.3%), and proximity to healthcare facilities (38.3%). Furthermore, 42.8% of participants preferred a physician with a shared cultural background, emphasizing the importance of culturally sensitive communication in clinical research.

Only 27.9% of participants reported a strong understanding of how clinical trials operate. Many respondents were either neutral (43.3%) or uncertain (28.9%), indicating a knowledge gap that may contribute to hesitancy.

Participants exhibited varied levels of knowledge about clinical trials. The highest awareness was associated with the statements: “Clinical trials may result in effective cancer treatments” (mean score: 0.78 ± 0.29) and “Clinical trials are research that may result in new drugs with fewer side effects” (mean score: 0.76 ± 0.30). Despite this, significant misconceptions persisted. Only 27.9% of participants believed that clinical trials are research that should be conducted on animals but are instead performed on humans. Furthermore, 79.6% were unaware of clinical trials being conducted in Arab countries, and 51.3% were unaware that informed consent is required before enrollment in clinical trials. More detailed information is presented in Supplementary Materials.

**Figure 3:**
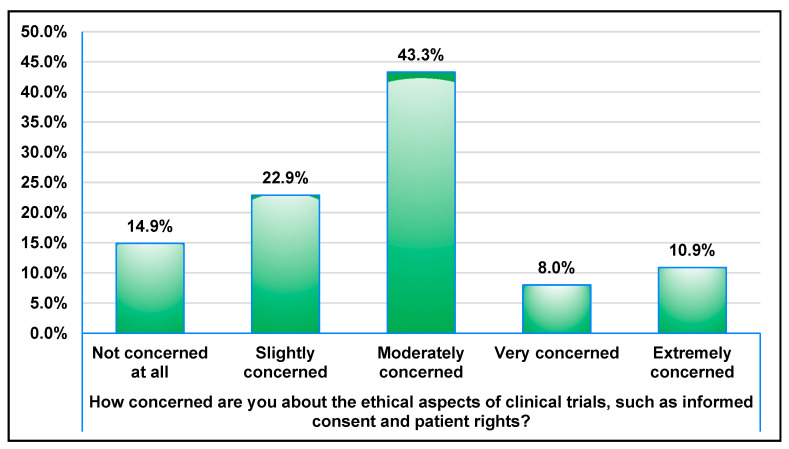
Distribution of the participants regarding concern about ethical issues of clinical trials.

Most participants (61.7%) believed that clinical trials play a significant role in advancing medical science, with 37.8% agreeing and 23.9% strongly agreeing. However, 31.3% remained neutral. Ethical concerns, such as informed consent and patient rights, were prominent among respondents. While 43.3% were moderately concerned, 10.9% expressed extreme concern, and 8.0% were very concerned, as shown in Figure 3.

Healthcare providers were the primary source of information (50.2%), followed by social media (30.3%), support groups (9.0%), and friends or family (8.5%). A small percentage of participants (1.0%) reported never searching for information about clinical trials. Although only 5.5% of participants had previous clinical trial experience, 35.3% were willing to enroll if recommended by a physician, and 25.9% expressed openness to participating in Phase I clinical trials. However, 20.4% explicitly stated they would decline participation. When asked about preferred modes of receiving clinical trial information, participants favored in-person consultations (62.7%), followed by social media (47.8%), educational seminars (45.3%), online videos (34.3%), and printed brochures (24.9%). A support system played a key role in clinical trial decision-making for 52.7% of participants who reported having guidance from family, friends, or support groups. However, 16.4% lacked a support network, and 30.8% were unsure.

### 3.2. Barriers to Participation in Clinical Trials

Table 2 provides information about the potential barriers reported by the participants. As shown in Table 2, fear of side effects, experimental treatments, and insufficient information emerged as the most frequently reported barriers. Additionally, 41.3% feared receiving a placebo, and 24.4% reported mistrust in healthcare providers. Other factors included concerns about privacy (23.9%), financial constraints (18.9%), time limitations (10.4%), and cultural or familial beliefs (9.0%).

### 3.3. Factors Influencing Perception, Willingness to Participate, and Awareness of Clinical Trials

This study examined the relationship between gender and perceptions of clinical trials among cancer patients, highlighting notable gender-based differences, as illustrated in Supplementary Table S1. Female participants were significantly more likely to perceive clinical trials as beneficial (*p* = 0.039). In contrast, male participants demonstrated a greater willingness to participate if recommended by a healthcare provider (*p* = 0.015) and in Phase I trials (*p* = 0.046). No significant gender differences were observed in awareness of clinical trials, trust in physicians, or concerns about ethical considerations. Furthermore, higher family income was positively associated with favorable perceptions of clinical trials (*p* = 0.038), while age and geographic location significantly influenced perceptions of risk (*p* = 0.012 and *p* = 0.037, respectively). Additional details and supporting data are available in Supplementary Materials.

## 4. Discussion

This study offers valuable insights into the awareness, perceptions, and willingness of Saudi cancer patients to participate in clinical trials. While 51.2% of participants reported awareness of clinical trials, only 27.9% demonstrated a strong understanding of how these trials function, highlighting a significant knowledge gap. Although some studies have reported higher awareness rates such as 77.5%, these figures often reflect general population samples or different subgroups [4,8]. Similarly, a study in Jordan found that 65% of cancer patients were unaware of clinical trials, further emphasizing the regional trend of limited knowledge [15]. (Healthcare providers emerged as the primary and most trusted source of information about clinical trials (50.2%), followed by social media (30.3%). This finding aligns with prior research, which reported that 73.6% of participants trusted healthcare providers for clinical trial-related information [16]. The notable reliance on social media suggests its growing influence in health communication. However, social media can enhance awareness, as reported by 81.4% of participants in another study, and it also raises concerns about the potential spread of misinformation [17]. To mitigate these risks, we recommend strengthening digital health literacy among patients and encouraging healthcare institutions and professionals to create or endorse trustworthy online content. Partnering with verified social media influencers in the healthcare field may also help improve message accuracy and reach. Most participants (57.2%) perceived clinical trials as beneficial, aligning with findings from other regions. For instance, a study in the United States reported that 67% of cancer patients held positive views toward clinical trials. However, the majority did not consider clinical trials as a first-line treatment option, opting to participate only if standard treatments failed or if no alternatives were available [18].

Despite these positive perceptions, concerns about potential risks remained significant, with 44.8% of participants in our study expressing apprehension. Similar concerns have been observed in the United States, where fear of company-sponsored trials and early-phase (Phase I) trials were prominent barriers [18]. Many of these fears stem from psychological risk perception rather than objective evidence. Providing empathetic, patient-centered education, peer stories from trial participants, and open dialogue with clinicians can alleviate anxiety and build trust in the trial process. Additional barriers identified in our study included misconceptions, lack of knowledge and awareness, fear of treatment and side effects, limited access to trusted sources of information, influence from friends and family, and challenges related to healthcare facility accessibility and proximity. Cultural norms and family dynamics also play a pivotal role in shaping patients’ decisions about clinical trial participation. Involving family members in decision-making, offering culturally tailored communication materials, and engaging religious or community leaders can improve patient comfort and willingness to enroll. Overcoming these hurdles requires enhanced patient education and awareness, training healthcare providers to foster research literacy, establishing patient support services, and offering financial and logistical assistance. Physician communication plays a critical role in trial participation. Structured discussions addressing trial benefits, risks, and common misconceptions, delivered in a culturally sensitive manner, may further support patient decision-making. Additionally, strengthening patient advocacy efforts can further promote informed decision-making and improve clinical trial participation rates. Although our study included participants from various provinces, we did not perform a region-specific analysis due to sample size limitations. Although our findings provide valuable insights, the reduced sample size compared to the initially calculated target may limit the generalizability and statistical power of the results, particularly for subgroup analyses. However, noticeable regional representation differences suggest the importance of tailoring awareness campaigns to regional sociocultural dynamics. The reliance on an online survey may have excluded individuals with limited digital access or lower digital literacy, which could affect the representativeness of the sample. While this study provides important quantitative insights, future research could incorporate qualitative methods, such as interviews or focus groups, to explore patients’ underlying motivations, fears, and perceptions in greater depth. Qualitative exploration would provide richer contextual understanding of the barriers identified and inform more targeted educational and policy interventions. Future studies should examine these differences more closely to support equitable trial access.

## 5. Conclusions

This study underscores the importance of improving awareness and trust in clinical trials among Saudi cancer patients. Misconceptions, logistical challenges, and cultural concerns remain significant barriers to participation. Providing accessible, reliable information and targeted educational initiatives can help bridge this gap, encouraging more patients to consider enrollment. Moving forward, healthcare providers should be equipped with tools to deliver patient-centered education about clinical trials. Culturally tailored communication strategies and digital outreach can help address misconceptions and improve engagement. Future research should explore the effectiveness of such interventions, assess region-specific barriers, and include broader, more diverse patient populations to improve generalizability. A national framework that integrates clinical trial awareness into routine cancer care could play a key role in improving participation rates across Saudi Arabia.

## Figures and Tables

**Table 1:** Demographic characteristics.

Items	Studied Participants (N = 201)	
	N	%
Age		
Mean± SD	39.51± 14.86	
Median (Range)	38.5 (14–83)	
≤18 years	11	5.5%
19–40 years	100	49.8%
41–60 years	80	39.8%
>60 years	10	5.0%
Gender		
Female	139	69.2%
Male	62	30.8%
Marital status		
Married	108	53.7%
Single	65	32.3%
Divorced	16	8.0%
Widowed	9	4.5%
Prefer not to say	3	1.5%
Educational level		
Illiterate	8	4.0%
Primary School	14	7.0%
Secondary School	57	28.4%
Bachelor’s Degree	94	46.8%
Diploma	3	1.5%
Master’s Degree	14	7.0%
Doctorate	10	5.0%
Others	1	0.5%
Employment Status		
Employed full-time	64	31.8%
Employed part-time	10	5.0%
Freelancer	4	2.0%
Housewife	4	2.0%
Not working	4	2.0%
Retired	22	10.9%
Student	33	16.4%
Unemployed	60	29.9%
Monthly family income		
Less than 6000 SAR	63	31.3%
6000–15,000 SAR	68	33.8%
15,001–30,000 SAR	30	14.9%
30,001–50,000 SAR	6	3.0%
More than 50,000 SAR	1	0.5%
Prefer not to say	33	16.4%
Province		
Makkah province	83	41.3%
Riyadh province	56	27.9%
Northern province	33	16.4%
Eastern province	15	7.5%
Madinah province	7	3.5%
Southern province	7	3.5%
Type of cancer		
Breast cancer	65	32.3%
Lymphoma	36	17.9%
Colorectal cancer	15	7.5%
Renal carcinoma	6	3.0%
Prostate cancer	5	2.5%
Lung cancer	5	2.5%
Other cancer (Not specified)	69	34.3%

SD: standard deviation.

**Table 2:** Barriers to participation among the studied participants.

	Studied Participants (N = 201)	
	No	%
What potential barriers would prevent you from participating in a clinical trial?		
Fear of side effects	166	82.6%
Fear of experimental treatment	115	57.2%
Lack of information	86	42.8%
Fear of receiving a placebo	83	41.3%
Lack of trust in healthcare providers	49	24.4%
Concerns about privacy	48	23.9%
Financial constraints	38	18.9%
Time constraints	21	10.4%
Family or cultural beliefs	18	9.0%

## Data Availability

Upon a justified request, the corresponding author can share the data.

## Supplementary Materials

The following supporting information is available online at: https://www.mdpi.com/article/10.3390/healthcare13091044/s1. Table S1 presents knowledge of clinical trials among cancer patients, including frequency, percentage, and overall knowledge scores. Table S2 shows associations between gender and perceptions related to clinical trials, such as trust in healthcare providers, perceived risks and benefits, and willingness to participate. Table S3 details the relationship between demographic variables and the perception of clinical trials as beneficial, using Mann–Whitney U and Kruskal–Wallis tests. Table S4 explores the relationship between demographic variables and the perception of clinical trials as risky. Table S5 includes the full bilingual (Arabic–English) version of the survey instrument used in the study, covering all sections: awareness, perceptions, trust, ethical concerns, participation willingness, barriers, and expectations. The English version of the full supplementary content is provided in the file “Supplementary Tables.docx.”
