# Supplementary material for: Clinical Trial Awareness, Perceptions, and Participation Among Cancer Patients in Saudi Arabia
Source: Healthcare (Basel). 2025 May 1;13(9):1044. doi: 10.3390/healthcare13091044 (PMC12071313; doi:10.3390/healthcare13091044)
Supplement: Supplementary file 1 [file healthcare-13-01044-s001.zip › healthcare-3554053-supplementary.pdf]

Supplementary tables

Table S1: Knowledge of clinical trials among cancer patients.

N			Studied participants			
			Frequency (N)	Percentage (%)	Mean	±SD
1	Clinical trials are research that should be done on animals but instead are done on humans.	No	57	28.4%	0.50	±0.38
		Don't know	88	43.8%		
		Yes	56	27.9%		
2	Clinical trials are research of drugs found safe on animals and now being tried on humans.	No	22	10.9%	0.70	±0.34
		Don't know	78	38.8%		
		Yes	101	50.2%		
3	Clinical trials are research on new drugs only.	No	65	32.3%	0.46	±0.37
		Don't know	88	43.8%		
		Yes	48	23.9%		
4	Clinical trials are research on new and old drugs.	No	37	18.4%	0.60	±0.37
		Don't know	86	42.8%		
		Yes	78	38.8%		
5	Clinical trials are research that may result in effective drugs against cancer	No	9	4.5%	0.78	±0.29
		Don't know	71	35.3%		
		Yes	121	60.2%		
6	Clinical trials are research that may result in new drugs with fewer side effects	No	12	6.0%	0.76	±0.30
		Don't know	73	36.3%		
		Yes	116	57.7%		
7	Medicine can advance without clinical trials	No	96	47.8%	0.35	±0.37
		Don't know	71	35.3%		
		Yes	34	16.9%		
8	Advanced cancer centers do not do a lot of clinical trials	No	49	24.4%	0.47	±0.32
		Don't know	116	57.7%		
		Yes	36	17.9%		
9	There are no clinical trials conducted in the Arab countries	No	41	20.4%	0.44	±0.26
		Don't know	144	71.6%		
		Yes	16	8.0%		
10	Clinical trials in Arab countries are not under any supervision	No	80	39.8%	0.34	±0.30
		Don't know	107	53.2%		
		Yes	14	7.0%		
11	Many patients are included in clinical trials without their consent	No	98	48.8%	0.30	±0.32
		Don't know	85	42.3%		
		Yes	18	9.0%		
12	It is okay to do clinical trials on patients by taking family consent only	No	111	55.2%	0.31	±0.38
		Don't know	57	28.4%		
		Yes	33	16.4%		
13	It is okay to do clinical trials on illiterate patients	No	124	61.7%	0.25	±0.35
		Don't know	53	26.4%		
		Yes	24	11.9%		
Total score					6.24	±1.83

Table S2: Associations between gender and categorical variables.

Item	Response	Females (N=139)	Males (N=62)	P-value
**Are you aware of the existence and purpose of clinical trials for cancer treatment?**	Not sure	24 (17.3%)	8 (12.9%)	0.781
	No	45 (32.4%)	21 (33.9%)	
	Yes	70 (50.4%)	33 (53.2%)	
**Do you perceive clinical trials as potentially beneficial for cancer patients?**	Neutral	58 (41.7%)	17 (27.4%)	0.039*
	Disagree	5 (3.6%)	5 (8.1%)	
	Strongly disagree	1 (0.7%)	0 (0.0%)	
	Agree	53 (38.1%)	21 (33.9%)	
	Strongly agree	22 (15.8%)	19 (30.6%)	
**Do you perceive clinical trials as potentially risky for cancer patients?**	Neutral	66 (47.5%)	22 (35.5%)	0.471
	Disagree	9 (6.5%)	7 (11.3%)	
	Strongly disagree	5 (3.6%)	2 (3.2%)	
	Agree	38 (27.3%)	22 (35.5%)	
	Strongly agree	21 (15.1%)	9 (14.5%)	
**If your physician recommends you to participate in a cancer clinical trial, do you trust that he or she would fully explain it to you?**	Not sure	51 (36.7%)	21 (33.9%)	0.796
	No	21 (15.1%)	8 (12.9%)	
	Yes	67 (48.2%)	33 (53.2%)	

**Do you believe you can freely ask your physician any questions about cancer clinical trials?**	Not sure	26 (18.7%)	8 (12.9%)	0.477
	No	9 (6.5%)	6 (9.7%)	
	Yes	104 (74.8%)	48 (77.4%)	
**Do you have a good understanding of how clinical trials work?**	Neutral	31 (22.3%)	15 (24.2%)	0.067
	Disagree	6 (4.3%)	4 (6.5%)	
	Strongly disagree	69 (49.6%)	18 (29.0%)	
	Agree	22 (15.8%)	17 (27.4%)	
	Strongly agree	11 (7.9%)	8 (12.9%)	
**Do you feel like clinical trials make a significant contribution to science?**	Neutral	48 (34.5%)	15 (24.2%)	0.333
	Disagree	4 (2.9%)	2 (3.2%)	
	Strongly disagree	7 (5.0%)	1 (1.6%)	
	Agree	47 (33.8%)	29 (46.8%)	
	Strongly agree	33 (23.7%)	15 (24.2%)	
**How concerned are you about the ethical aspects of clinical trials, such as informed consent and patient rights?**	Not concerned at all	21 (15.1%)	9 (14.5%)	0.701
	Moderately concerned	63 (45.3%)	24 (38.7%)	
	Very concerned	12 (8.6%)	4 (6.5%)	
	Slightly concerned	28 (20.1%)	18 (29.0%)	

	Extremely concerned	15 (10.8%)	7 (11.3%)	
**Have you ever participated in a clinical trial before?**	No	132 (95.0%)	58 (93.5%)	0.74
	Yes	7 (5.0%)	4 (6.5%)	
**Would you be willing to participate in a clinical trial if recommended by your healthcare provider?**	Not sure	68 (48.9%)	21 (33.9%)	0.015*
	No	31 (22.3%)	10 (16.1%)	
	Yes	40 (28.8%)	31 (50.0%)	
**Would you be willing to participate in a Phase I clinical trial?**	Not sure	62 (44.6%)	20 (32.3%)	0.046*
	No	48 (34.5%)	19 (30.6%)	
	Yes	29 (20.9%)	23 (37.1%)	
**Do you have access to a support system (family, friends, support groups) to help you make decisions about participating in a clinical trial?**	Not sure	48 (34.5%)	14 (22.6%)	0.081
	No	25 (18.0%)	8 (12.9%)	
	Yes	66 (47.5%)	40 (64.5%)	

*P value< 0.05 is significant, **P value< 0.01 is significant, analysis done by Chi-Square Test, Fischer Exact test and Monte-Carlo correction

Table S3: Relation between perceptions of clinical trial as potentially beneficial for cancer patients with demographic data.

Items		Perceptions score			Test value	P-value
		Median	IQR			
**Gender**	Female	4.0	3.0	4.0	Z_{MWU}= 1.770	0.077
	Male	4.0	3.0	5.0		
**Age**	≤18 years	4.0	3.0	4.0	Kw= 0.426	0.935
	19-40 years	4.0	3.0	4.0		
	41- 60 years	4.0	3.0	4.0		
	>60 years	3.0	3.0	5.0		

**Marital Status**	Divorced	3.0	3.0	4.0	Kw= 7.480	0.113
	Married	4.0	3.0	4.0		
	Prefer not to say	3.0	3.0	3.0		
	Single	4.0	3.0	5.0		
	Widowed	3.0	3.0	4.0		
**Educational level**	Baby	5.0	5.0	5.0	Kw= 11.58	0.115
	Bachelor's Degree	4.0	3.0	4.0		
	Diploma	3.0	3.0	5.0		
	Doctorate	4.0	4.0	5.0		
	Illiterate	3.5	3.0	4.0		
	Master's Degree	3.5	3.0	4.0		
	Primary School	3.0	3.0	4.0		
	Secondary School	4.0	3.0	4.0		
**Employment Status**	Employed full-time	4.0	3.0	4.0	Kw= 10.216	0.177
	Employed part-time	3.5	3.0	4.0		
	Freelancer	3.0	3.0	3.0		
	Housewife	3.0	3.0	3.5		
	Not working	4.5	4.0	5.0		
	Retired	4.0	3.0	4.0		
	Student	4.0	3.0	4.0		
	Unemployed	4.0	3.0	4.0		
**Monthly family income**	15,001 - 30,000 SAR	4.0	3.0	5.0	Kw= 11.76	**0.038***
	30,001 - 50,000 SAR	5.0	4.0	5.0		
	6000 - 15,000 SAR	4.0	3.0	4.0		
	Less than 6000 SAR	3.0	3.0	4.0		
	Prefer not to say	4.0	3.0	4.0		
	More than 50,000 SAR	5.0	5.0	5.0		
**The province**	Eastern province	3.0	3.0	4.0	Kw= 8.024	0.155
	Madinah province	4.0	3.0	5.0		
	Makkah province	4.0	3.0	4.0		
	Northen province	3.0	3.0	4.0		
	Riyadh province	4.0	3.0	5.0		
	Southern province	4.0	4.0	4.0		
**Type of Cancer (if applicable)**	Breast cancer	4.0	3.0	4.0	Kw= 5.102	0.531
	Colorectal cancer	4.0	3.0	4.0		
	Lung cancer	4.0	4.0	4.0		
	Lymphoma	4.0	3.0	5.0		
	Other cancer (Not specified)	4.0	3.0	4.0		
	Prostate cancer	4.0	3.0	4.0		
	Renal carcinoma	4.0	3.0	4.0		

P value< 0.05 is significant, analysis done by Mann-Whitney U test and Kruskal Wallis Test

Table S4: Relation between perceptions as potentially risky for cancer patients with demographic data.

Items		Perceptions score			Test value	P-value
		Mean	IQR			
**Gender**	Female	3.0	3.0	4.0	Z_{MWU}= 0.365	0.715
	Male	3.5	3.0	4.0		
**Age**	≤18 years	3.0	2.0	3.0	Kw= 10.904	**0.012***
	19-40 years	3.0	3.0	4.0		
	41- 60 years	4.0	3.0	4.0		
	>60 years	3.0	3.0	3.0		
**Marital Status**	Divorced	3.0	3.0	4.0	Kw= 1.811	0.771
	Married	3.0	3.0	4.0		
	Prefer not to say	3.0	3.0	5.0		
	Single	3.0	3.0	4.0		
	Widowed	3.0	3.0	4.0		
**Educational level**	Baby	2.0	2.0	2.0	Kw= 10.87	0.144
	Bachelor's Degree	3.5	3.0	4.0		
	Diploma	3.0	1.0	4.0		
	Doctorate	4.5	3.0	5.0		
	Illiterate	3.0	3.0	4.0		
	Master's Degree	4.0	3.0	4.0		
	Primary School	3.0	3.0	4.0		
	Secondary School	3.0	3.0	4.0		
**Employment Status**	Employed full-time	4.0	3.0	4.5	Kw= 11.53	0.117
	Employed part-time	4.0	3.0	4.0		
	Freelancer	3.0	3.0	3.5		
	Housewife	3.0	3.0	3.5		
	Not working	2.5	1.5	3.5		
	Retired	3.0	3.0	4.0		
	Student	4.0	3.0	4.0		
	Unemployed	3.0	3.0	4.0		
**Monthly family income**	15,001 - 30,000 SAR	4.0	3.0	4.0	Kw= 9.99	0.075
	30,001 - 50,000 SAR	4.5	3.0	5.0		
	6000 - 15,000 SAR	3.5	3.0	4.0		
	Less than 6000 SAR	3.0	3.0	4.0		
	Prefer not to say	3.0	3.0	4.0		
	More than 50,000 SAR	4.0	4.0	4.0		
**The province**	Eastern province	4.0	3.0	4.0	Kw=	**0.037***

	Madinah province	4.0	3.0	5.0	11.86	
	Makkah province	3.0	3.0	4.0		
	Northen province	3.0	3.0	4.0		
	Riyadh province	3.0	3.0	4.0		
	Southern province	4.0	3.0	4.0		
**Type of Cancer (if applicable)**	Breast cancer	3.0	3.0	4.0	Kw= 11.51	0.084
	Colorectal cancer	3.0	2.0	4.0		
	Lung cancer	3.0	3.0	4.0		
	Lymphoma	4.0	3.0	4.0		
	Other cancer (Not specified)	3.0	3.0	4.0		
	Prostate cancer	4.0	3.0	4.0		
	Renal carcinoma	4.5	3.0	5.0		

P value< 0.05 is significant, analysis done by Mann-Whitney U test and Kruskal Wallis Test

Table S5

Translated Survey

استبيان يهدف لقياس مدى وعي مرضى السرطان في المملكة العربية السعودية بالتجارب السريرية، وتصوراتهم بشأن فوائدها والاستعداد للمشاركة فيها.

أشكركم على مشاركتكم في دراستنا البحثية عن وعي مرضى السرطان بالتجارب السريرية، والتصورات بشأن فوائدها ومخاطرها، والاستعداد للمشاركة فيها. ستساهم أفكاركم القيِّمة في تحسين علاج السرطان والبحوث في المملكة العربية السعودية.

المشاركة طوعية، فإن وافقتم ستحتاجون إلى استكمال هذا الاستبيان، الذي لا ينبغي أن يستغرق أكثر من 5-10 دقائق من وقتكم.

للاستفسار يُرجى التواصل عبر الايميل:

شكرا لكم على مساهمتكم القيِّمة

A survey about Cancer Patients' Awareness of Clinical Trials, Perceptions on the Benefit, and Willingness to Participate - Saudi Perspectives

Thank you for participating in our research study on cancer patients' awareness of clinical trials, their perceptions of the benefits and risks, and their willingness to participate in Saudi Arabia. Your valuable insights will contribute to improving cancer treatment and research in the region.

Participation is voluntary; if you agree, you will need to complete this survey, which should not take more than 5-10 minutes of your time.

If you have any questions about the survey, please email us:

Thank you for your valuable contribution

**1**	**Gender:**<br>• Male<br>• Female	**الجنس:**<br>• ذكر<br>• أنثى
**2**	**Age:**	العمر:
3	**Marital Status:**<br>• Single<br>• Married<br>• Divorced<br>• Widowed<br>• Prefer not to say	**الحالة الاجتماعية:**<br>• أعزب<br>• متزوج<br>• مُطلّق<br>• أرمل<br>• افضّل عدم الإجابة
4	**Education Level:**<br>• Primary School<br>• Secondary School<br>• Bachelor's Degree<br>• Master's Degree<br>• Doctorate<br>• Other (please specify): ________	**مستوى التعليم:**<br>• ابتدائي<br>• ثانوي<br>• بكالوريوس<br>• ماجيستير<br>• دكتوراه<br>• غير ذلك (يرجى التحديد): ___________
5	**Employment Status:**<br>• Employed full-time<br>• Employed part-time<br>• Unemployed<br>• Student<br>• Retired<br>• Other (please specify):___________	**الحالة الوظيفية:**<br>• دوام كامل<br>• دوام جزئي<br>• غير موظف<br>• طالب<br>• متقاعد<br>• أخرى (يرجى التحديد):___________
6	**monthly Household Income:**<br>• Less than 6000 SAR<br>• 6000 - 15,000 SAR<br>• 15,001 - 30,000 SAR<br>• 30,001 - 50,000 SAR<br>• Over 50,000 SAR<br>• Prefer not to say	**دخل الأسرة الشهري:**<br>• أقل من 6000 ريال سعودي<br>• 6000 – 15,000 ريال سعودي<br>• 15,001 – 30,000 ريال سعودي<br>• 30,001 – 50,000 ريال سعودي<br>• أكثر من 50,000 ريال سعودي<br>• افضّل عدم الإجابة
7	**The province:**<br>• Riyadh province<br>• Makkah province<br>• The Eastern province<br>• Other (please specify): ____________	**المنطقة:**<br>• منطقة الرياض<br>• منطقة مكة المكرمة<br>• المنطقة الشرقية<br>• أخرى (يُرجى التحديد): ___________
8	**Type of Cancer (if applicable):**<br>• Breast<br>• Lung<br>• Prostate<br>• Colorectal	**نوع السرطان (إن وجد):**<br>• الثدي<br>• الرئة<br>• البروستاتا<br>• القولون والمستقيم

	• Other (please specify): __________	• أخرى (يرجى التحديد): _____________

Section A: Demographic Information **المعلومات الشخصية**

Section B: Awareness of Clinical Trials **الوعي بالتجارب السريرية**

9	**Are you aware of the existence and purpose of clinical trials for cancer treatment?**<br>• Yes<br>• No<br>• Not sure	**هل أنت على علم بوجود التجارب السريرية لعلاج السرطان وماهو الغرض منها؟**<br>• نعم<br>• لا<br>• غير متأكد
10	**If yes, how did you become aware of clinical trials?**<br>• Healthcare provider<br>• Family or friends<br>• Social media<br>• Other (please specify):_________	**ان كانت الإجابة على السؤال السابق بنعم، كيف أصبحت على علم بوجود وأهمية التجارب السريرية؟**<br>• مقدّم الرعاية الصحية<br>• العائلة أو الأصدقاء<br>• وسائل التواصل الاجتماعي<br>• أخرى (يرجى التحديد):_________

Section C: Perceptions of Clinical Trials **التصورات بشأن التجارب السريرية**

11	**Do you perceive clinical trials as potentially beneficial for cancer patients?**<br>• Strongly Disagree<br>• Disagree<br>• Neutral<br>• Agree<br>• Strongly Agree	**هل ترى أن التجارب السريرية قد تكون مفيدة لمرضى السرطان؟**<br>• لا أوافق بشدة<br>• غير موافق<br>• حيادي<br>• موافق<br>• موافق بشدة
12	**Do you perceive clinical trials as potentially risky for cancer patients?**<br>• Strongly Disagree<br>• Disagree<br>• Neutral<br>• Agree<br>• Strongly Agree	**هل ترى أن التجارب السريرية قد تكون محفوفة بالمخاطر بالنسبة لمرضى السرطان؟**<br>• لا أوافق بشدة<br>• غير موافق<br>• حيادي<br>• موافق<br>• موافق بشدة

Section D: Trust in Healthcare Providers **الثقة بمقدميّ الرعاية الصحية**

13	**If your physician recommends you to participate in a cancer clinical trial, do you trust that he or she would fully explain it to you?**	**إذا أوصى طبيبك بالمشاركة في تجربة سريرية للسرطان، فهل تثق بأنه سيشرح لك ذلك بشكل كامل؟**<br>• نعم<br>• لا

	• Yes<br>• No<br>• Not sure	• غير متأكد
14	**Do you believe you can freely ask your physician any questions about cancer clinical trials?**<br>• Yes<br>• No<br>• Not sure	**هل تعتقد أنه يمكنك أن تسأل طبيبك بحرية أي أسئلة حول التجارب السريرية للسرطان؟**<br>• نعم<br>• لا<br>• غير متأكد

Section E: Factors Influencing Participation **العوامل المؤثرة على المشاركة**

15	**How important are the following factors in influencing your decision to participate in a clinical trial? (Rate each factor on a scale of 1 to 5, with 1 being "Not Important" and 5 being "Very Important."):**<br>• Proximity to your residence<br>• Your physician's recommendation<br>• Potential side effects of the treatment<br>• Access to information about the trial<br>• The trial's potential to benefit cancer research<br>• Previous knowledge of clinical trials<br>• Financial compensation<br>• Family support<br>• Fear of receiving a placebo<br>• Fear of experimental treatment<br>• Concerns about privacy	**ما مدى أهمية العوامل التالية في التأثير على قرارك بالمشاركة في تجربة سريرية؟ (قم بتقييم كل عامل من 1 إلى 5، حيث يشير الرقم 1 إلى "غير مهم" والرقم 5 إلى "مهم جدًا"):**<br>• القرب من مكان إقامتك<br>• توصية طبيبك<br>• الآثار الجانبية المحتملة للعلاج<br>• الوصول إلى المعلومات حول التجربة السريرية<br>• الفائدة المرجوة من التجربة السريرية<br>• المعرفة السابقة بالتجارب السريرية<br>• تعويضات مالية<br>• الدعم الأسري<br>• الخوف من تلقي العلاج الوهمي<br>• الخوف من العلاج التجريبي<br>• مخاوف بشأن الخصوصية

Section F: Expectations from Clinical Trials **التوقعات حول التجارب السريرية**

16	**What features would you expect from a good cancer clinical trial program (select all that apply)?**<br>• Providing me with educational information about clinical trials<br>• Accessible online information about the program<br>• Offer flexible schedules<br>• Close to where I live<br>• A physician with a similar cultural background as me<br>• Others (please specify):_______________	**ما هي بعض الميزات التي تتوقعها من برنامج التجارب السريرية الجيد (يمكن اختيار أكثر من اجابة)؟**<br><br>• تزويدي بالمعلومات التثقيفية حول التجارب السريرية<br>• وجود مواقع الكترونية حول البرنامج<br>• تقديم جداول زمنية مرنة<br>• قريب من مكان اقامتي<br>• ثقافة الطبيب قريبة او شبيهة بثقافتي<br>أخرى (يرجى التحديد): _______________

18	**Clinical trials are research that should be done on animals but instead are done on humans.**<br>• Yes<br>• No<br>• Don't know	التجارب السريرية هي أبحاث يجب إجراؤها على الحيوانات، ولكن بدلاً من ذلك يتم إجراؤها على البشر.<br>1- نعم<br>2-لا<br>3- لا أعلم
19	**Clinical trials are research of drugs found safe on animals and now being tried on humans.**<br>• Yes<br>• No<br>• Don't know	التجارب السريرية هي أبحاث حول أدوية وُجد أنها آمنة على الحيوانات ويتم تجربتها الآن على البشر.<br>1- نعم<br>2-لا<br>3- لا أعلم
20	**Clinical trials are research on new drugs only.**<br>• Yes<br>• No<br>• Don't know	التجارب السريرية هي أبحاث على أدوية جديدة فقط.<br>1- نعم<br>2-لا<br>3- لا أعلم
21	**Clinical trials are research on new and old drugs.**<br>• Yes<br>• No<br>• Don't know	التجارب السريرية هي أبحاث على الأدوية الجديدة والقديمة.<br>1- نعم<br>2-لا<br>3- لا أعلم
22	**Clinical trials are research that may result in effective drugs against cancer.**<br>• Yes<br>• No<br>• Don't know	التجارب السريرية هي الأبحاث التي قد تؤدي إلى أدوية فعالة ضد السرطان.<br>1- نعم<br>2-لا<br>3- لا أعلم
23	**Clinical trials are research that may result in new drugs with fewer side effects.**<br>• Yes<br>• No<br>• Don't know	التجارب السريرية هي الأبحاث التي قد تؤدي إلى أدوية جديدة ذات آثار جانبية أقل.<br>1- نعم<br>2-لا<br>3- لا أعلم
24	**Medicine can advance without clinical trials.**<br>• Yes<br>• No<br>• Don't know	يمكن للطب أن يتقدم دون تجارب سريرية.<br>1- نعم<br>2-لا<br>3- لا أعلم
25	**Advanced cancer centers do not do a lot of clinical trials.**<br>• Yes<br>• No<br>• Don't know	مراكز الأورام المتطورة لا تقوم بالكثير من التجارب السريرية.<br>1- نعم<br>2-لا<br>3- لا أعلم
26	**There are no clinical trials conducted in the Arab countries.**<br>• Yes<br>• No<br>• Don't know	لا توجد تجارب سريرية أجريت في الدول العربية.<br>1- نعم<br>2-لا<br>3- لا أعلم
27	**Clinical trials in Arab countries are not under any supervision.**	التجارب السريرية في الدول العربية لا تخضع لأي إشراف.

	• Yes<br>• No<br>• Don’t know	1- نعم<br>2-لا<br>3- لا أعلم
28	**Many patients are included in clinical trials without their consent.**<br>• Yes<br>• No<br>• Don’t know	**يتم إدراج العديد من المرضى في التجارب السريرية دون موافقتهم.**<br>1- نعم<br>2-لا<br>3- لا أعلم
29	**It is okay to do clinical trials on patients by taking family consent only.**<br>• Yes<br>• No<br>• Don’t know	**لا بأس بإجراء التجارب السريرية على المرضى بأخذ موافقة الأسرة فقط.**<br>1- نعم<br>2-لا<br>3- لا أعلم
30	**It is okay to do clinical trials on illiterate patients.**<br>• Yes<br>• No<br>• Don’t know	**لا بأس بإجراء التجارب السريرية على المرضى الأميين.**<br>1- نعم<br>2-لا<br>3- لا أعلم

Section G: Understanding of Clinical Trials **فهم التجارب السريرية**

17	**have a good understanding of how clinical trials work.**<br>• Strongly Disagree<br>• Disagree<br>• Neutral<br>• Agree<br>• Strongly Agree	**لديك فهم جيد لكيفية عمل التجارب السريرية.**<br>• لا أوافق بشدة<br>• غير موافق<br>• حيادي<br>• موافق<br>• موافق بشدة

Section H: Knowledge of Clinical trials **المعرفة بالتجارب السريرية**

Section I: Contribution to Science **مساهمة التجارب السريرية في العلوم**

31	**Do you feel like clinical trials make a significant contribution to science?**<br>• Strongly Disagree<br>• Disagree<br>• Neutral	**هل تشعر أن التجارب السريرية تقدم مساهمة كبيرة في العلوم؟**<br>• لا أوافق بشدة<br>• غير موافق<br>• حيادي

	• Agree<br>• Strongly Agree	• موافق<br>• موافق بشدة

Section J: Ethical **الاعتبارات الأخلاقية**

32	**How concerned are you about the ethical aspects of clinical trials, such as informed consent and patient rights?**<br>• Not concerned at all<br>• Slightly concerned<br>• Moderately concerned<br>• Very concerned<br>• Extremely concerned	**ما مدى قلقك بشأن الجوانب الأخلاقية للتجارب السريرية، مثل الموافقة المستنيرة (المسبقة) وحقوق المريض؟**<br>• غير قلق على الاطلاق<br>• قلق قليلاً<br>• قلق إلى حد ما<br>• قلق جدا<br>• قلق للغاية

Section K: Previous Experience with Clinical Trials

33	**Have you ever participated in a clinical trial before?**<br>• Yes<br>• No	**هل سبق لك أن شاركت في تجربة سريرية؟**<br>• نعم<br>• لا
34	**If yes, please briefly describe your experience:**	**إذا كانت الإجابة بنعم، يرجى وصف تجربتك بإيجاز:**

Section L: Sources of Information **مصادر المعلومات**

35	**Where do you primarily seek information about cancer and clinical trials?**<br>• Healthcare providers<br>• Social media<br>• Friends and family<br>• Support groups<br>• Other (please specify): ______________	**أين تبحث في المقام الأول عن معلومات حول الأورام والتجارب السريرية؟**<br>• مقدمي الرعاية الصحية<br>• وسائل التواصل الاجتماعي<br>• الأصدقاء والعائلة<br>• مجموعات الدعم<br>• أخرى (يرجى التحديد): ____________

Section M: Willingness to Participate in Clinical Trials **الاستعداد للمشاركة في التجارب السريرية**

36	**Would you be willing to participate in a clinical trial if recommended by your healthcare provider?**<br>• Yes<br>• No<br>• Not sure	**هل ستكون على استعداد للمشاركة في تجربة سريرية إذا أوصى بها مقدم الرعاية الصحية الخاص بك؟**<br>• نعم<br>• لا<br>• غير متأكد

37	**Would you be willing to participate in a Phase I clinical trial?**<br>• Yes<br>• No<br>• Not sure	**هل أنت على استعداد للمشاركة في المرحلة الأولى من التجارب السريرية؟**<br>• نعم<br>• لا<br>• غير متأكد

Section N: Preferred Information Channels **مصادر المعلومات المفضلة**

38	**How would you prefer to receive information about clinical trials? (Select all that apply):**<br>• In-person consultations<br>• Printed brochures<br>• Educational seminars<br>• Online videos<br>• Social media<br>• Other (please specify): ________	**كيف تفضل الحصول على معلومات حول التجارب السريرية؟ (يُمكن اختيار أكثر من اجابة):**<br>• المشاورات الشخصية<br>• الكتيبات المطبوعة<br>• الندوات التثقيفية<br>• مقاطع فيديو<br>• وسائل التواصل الاجتماعي<br>• أخرى (يرجى التحديد): ________

Section O: Support Systems **نظام الدعم**

**39**	**Do you have access to a support system (family, friends, support groups) to help you make decisions about participating in a clinical trial?**<br>• Yes<br>• No<br>• Not sure	**هل يمكنك الوصول الى نظام دعم من (العائلة أو الأصدقاء أو من مجموعات الدعم) لمساعدتك في اتخاذ القرارات بشأن المشاركة في تجربة سريرية؟**<br>• نعم<br>• لا<br>• غير متأكد

Section P: Barriers to Participation **عوائق المشاركة**

40	**What potential barriers would prevent you from participating in a clinical trial? (Select all that apply):**<br>• Fear of side effects<br>• Fear of receiving a placebo<br>• Concerns about privacy<br>• Lack of information	**ما هي العوائق المحتملة التي قد تمنعك من المشاركة في تجربة سريرية؟ (يُمكن اختيار أكثر من إجابة):**<br>• الخوف من الآثار الجانبية<br>• الخوف من تلقي العلاج الوهمي<br>• مخاوف بشأن الخصوصية<br>• نقص المعلومات<br>• انعدام الثقة في مقدمي الرعاية الصحية<br>• معتقدات المجتمع الثقافية أو العائلية

	• Lack of trust in healthcare providers<br>• Family or cultural beliefs<br>• Time constraints<br>• Financial constraints<br>• Fear of experimental treatment<br>• Other (please specify): ________	ضيق الوقت •<br>قيود مالية •<br>الخوف من العلاج التجريبي •<br>أخرى (يرجى التحديد): ________ •

Section Q: Future Expectations **توقعات تحسينية**

41	**How can the healthcare system improve your experience with clinical trials?**	**كيف يمكن لنظام الرعاية الصحية تحسين تجربتك مع التجارب السريرية؟**

Section R: Additional Comments **تعليقات إضافية**

42	**Do you have any additional comments or insights you'd like to share about clinical trials and cancer treatment in Saudi Arabia?**	**هل لديك أي تعليقات أو أفكار إضافية ترغب في مشاركتها حول التجارب السريرية وعلاج السرطان في المملكة العربية السعودية؟**

نشكركم على وقتكم في استكمال هذا الاستبيان. سوف تساهم آراءكم في فهم أفضل لوجهات نظر مرضى السرطان بشأن التجارب السريرية في المملكة العربية السعودية وتساعد في تحسين رعاية مرضى السرطان في المنطقة.

Thank you for taking the time to complete this survey. Your responses will contribute to a better understanding of cancer patients' perspectives on clinical trials in Saudi Arabia and help improve cancer care in the region. Your input is invaluable.
